# High Prevalence of Drug Resistance and Class 1 Integrons in *Escherichia coli* Isolated From River Yamuna, India: A Serious Public Health Risk

**DOI:** 10.3389/fmicb.2021.621564

**Published:** 2021-02-09

**Authors:** Nambram Somendro Singh, Neelja Singhal, Manish Kumar, Jugsharan Singh Virdi

**Affiliations:** ^1^Department of Microbiology, University of Delhi South Campus, New Delhi, India; ^2^Department of Biophysics, University of Delhi South Campus, New Delhi, India

**Keywords:** commensal *E. coli*, plasmid-mediated antimicrobial resistance genes, integrons, multidrug resistance, horizontal transfer of genes

## Abstract

Globally, urban water bodies have emerged as an environmental reservoir of antimicrobial resistance (AMR) genes because resistant bacteria residing here might easily disseminate these traits to other waterborne pathogens. In the present study, we have investigated the AMR phenotypes, prevalent plasmid-mediated AMR genes, and integrons in commensal strains of *Escherichia coli*, the predominant fecal indicator bacteria isolated from a major urban river of northern India Yamuna. The genetic environment of *bla*_*CTX–M–*15_ was also investigated. Our results indicated that 57.5% of the *E. coli* strains were resistant to at least two antibiotic classes and 20% strains were multidrug resistant, i.e., resistant to three or more antibiotic classes. The multiple antibiotic resistance index of about one-third of the *E. coli* strains was quite high (>0.2), reflecting high contamination of river Yamuna with antibiotics. With regard to plasmid-mediated AMR genes, *bla*_*TEM–*1_ was present in 95% of the strains, followed by q*nr*S1 and *arm*A (17% each), *bla*_*CTX–M–*15_ (15%), *str*A*-str*B (12%), and *tet*A (7%). Contrary to the earlier reports where *bla*_*CTX–M–*15_ was mostly associated with pathogenic phylogroup B2, our study revealed that the CTX-M-15 type extended-spectrum β-lactamases (ESBLs) were present in the commensal phylogroups A and B1, also. The genetic organization of *bla*_*CTX–M–*15_ was similar to that reported for *E. coli*, isolated from other parts of the world; and IS*Ecp1* was present upstream of *bla*_*CTX–M–*15_. The integrons of classes 2 and 3 were absent, but class 1 integron gene *intI1* was present in 75% of the isolates, denoting its high prevalence in *E. coli* of river Yamuna. These evidences indicate that due to high prevalence of plasmid-mediated AMR genes and *intI1*, commensal *E. coli* can become vehicles for widespread dissemination of AMR in the environment. Thus, regular surveillance and management of urban rivers is necessary to curtail the spread of AMR and associated health risks.

## Introduction

The gastrointestinal tract of humans and animals is regarded as the primary/natural habitat of *Escherichia coli*. Besides its natural habitat, *E. coli* is also found in secondary habitats like aquatic and terrestrial reservoirs (Méric et al., 2013). Aquatic environments, especially urban water bodies, harbor a heterogeneous collection of microorganisms originating from fecal, hospital, agricultural, and veterinary sources. Moreover, several studies have suggested that aquatic environments serve as genetic reactors that promote transfer of antimicrobial resistance (AMR) and virulence genes among bacteria (Baquero et al., 2008; Hasegawa et al., 2018).

The phylogrouping methods commonly used for population/clonal studies of *E. coli* include multilocus sequence typing (MLST) (Aanensen and Spratt, 2005), multilocus enzyme electrophoresis (MLEE), triplex PCR, etc. Triplex PCR is a widely used rapid and simple technique for phylotyping *E. coli.* In triplex PCR, three genes (*chuA*, *yjaA*, and a gene encoding a fragment of a putative lipase esterase) are PCR-amplified (Clermont et al., 2000). Based on the presence or absence of these three genetic elements, a strain can be assigned to belong to any of the four phylogroups—A, B1, B2, and D (Clermont et al., 2000). Several researchers have studied AMR in *E. coli* isolated from aquatic environments and have reported that these traits are easily transmissible among bacterial species with the help of several mobile genetic elements like integrons, insertion sequences (ISs), plasmids, and transposons (Su et al., 2012; Koczura et al., 2013; Liebana et al., 2013; Pereira et al., 2013; Kaushik et al., 2018). Integrons are regarded as the primary vehicles that disseminate AMR genes among bacterial species (Gillings, 2014) because they can be located on conjugative plasmids, which enhance their horizontal spread.

River Yamuna is a major river of northern India, which is associated with several anthropogenic activities of the population residing in the National Capital Region of India. It gets contaminated with effluents originating from hospital and municipal wastewaters; discharge from livestock, poultry, and agriculture production plants; industries; etc. The high levels of pollutants are expected to provide a positive selection pressure for increasing AMR in bacterial population residing there (Kümmerer, 2009; Tacão et al., 2012). Thus, it is expected to be a crucial reservoir of a diverse *E. coli* populations and an ideal ecological niche for studying strains with diverse phenotypes, genotypes, and AMR. In an earlier study published from our laboratory, we had described the β-lactam susceptibilities and β-lactamase genes in 61 *E. coli* strains of all phylogroups (A, B1, B2, and D) isolated from river Yamuna (Bajaj et al., 2015). Though AMR resistance has been investigated for commensal phylogroups of *E. coli* isolated from veterinary or clinical sources, only a few studies have investigated the AMR phenotypes, genes, and integrons in commensal *E. coli* isolated from urban rivers. The commensal strains of *E. coli* in urban rivers can easily disseminate AMR determinants to pathogenic *E. coli* or other waterborne pathogens via mobile genetic elements. Thus, it is important to study the AMR determinants and integrons in commensal strains residing in water bodies also. Our study is the first report on AMR phenotypes, plasmid-mediated AMR genes, and integrons in the strains of commensal phylogroups (A and B1) of *E. coli* from river Yamuna, India.

## Materials and Methods

### Sample Processing and Isolation of *Escherichia coli*

Two hundred water samples were collected from different sites along the entire stretch of the river Yamuna, which flows through the National Capital Region of India in sterile screw-capped bottles, transported to the laboratory on ice, and processed within 6 h of the sample collection. A schematic figure showing the details of the sampling sites has been published earlier (Bajaj et al., 2015). Enrichment of the samples for isolation of *Escherichia coli* was performed using a published method (Ram et al., 2008). Briefly, 100 ml of water sample was filtered through a 0.45 μm membrane filter (Millipore, MA, United States). The membrane filter was cut into four pieces, and each piece was incubated in 50 ml of MacConkey broth at 37°C, 220 rpm, overnight. The next day, a loopful of the broth culture was streaked on the surface of MacConkey agar plates and incubated at 35°C for 18–20 h. One hundred sixty-two typical *E. coli* colonies were selected and maintained as pure cultures on Luria–Bertani (LB) agar slants at 4°C. Of these, 126 isolates were presumptively identified as *E. coli* using API 20E strips (bioMérieux, France). API 20E is a standardized kit of biochemical tests used to identify members of the family *Enterobacteriaceae* and other non-fastidious Gram-negative rods.

### Isolation of Genomic DNA, and PCR Amplification of Gene Encoding 16s rRNA and Phylogrouping Based on Triplex PCR

DNA was extracted from the *E. coli* strains using the boilinglysis method (Rodríguez-Baño et al., 2004). The gene encoding 16S rRNA was PCR-amplified using universal eubacterial forward primer 27F (5′AGAGTTTGATCCTGGCTCAG3′) and reverse primer 1492R (5′ACGGCTACCTTGTTACGACTT3′). The contents of the PCR mixture were 1 × PCR buffer (1.5 MgCl_2_, 1.5 mM of KCl, 10 mM of Tris–HCl, and 0.1% Triton X-100), 200 μM of the four dNTPs, 1 U of Taq DNA polymerase (New England Biolabs, Ipswich, MA, United States), 10 pmol of forward and reverse primers, and 1 ng of genomic DNA in a final volume of 25 μl. The PCR conditions and methods for purification of PCR amplicons and sequencing have been described earlier (Singhal et al., 2019). Briefly, the PCR amplicons were purified by HiYield^TM^ extraction kit (RBC Bioscience, New Taipei City, Taiwan) and sequenced at a commercial facility using Sanger sequencing (Invitrogen BioServices India Pvt. Ltd., Bangalore, India). The nucleotide sequence homology was analyzed using the nucleotide BLAST (BLASTn) algorithm available at the National Center for Biotechnology Information (NCBI).

The phylogenetic profiles of all the isolates were determined by triplex PCR (Clermont et al., 2000); and 40 strains representing the commensal phylogroups (A and B1) were selected for studying the AMR phenotypes, plasmid-mediated AMR genes, and integrons.

### Determining Antimicrobial Susceptibilities, and Extended-Spectrum β-Lactamase and AmpC Production

Antimicrobial susceptibilities of *E. coli* strains for various classes of antibiotics like β-lactams, aminoglycosides, quinolones, and tetracycline were determined by Kirby–Bauer disk diffusion method. The antibiotic disks (Himedia, India) that were used in this study were (charge in μg/disk) as follows: ampicillin (10 μg), piperacillin (100 μg), amoxicillin–clavulanic acid (20/10 μg), cefazolin (30 μg), cefuroxime (30 μg), cefotaxime (30 μg), cefepime (30 μg), streptomycin (10 μg), kanamycin (30 μg), tobramycin (10 μg), netilmicin (30 μg), amikacin (30 μg), nalidixic acid (30 μg), ciprofloxacin (5 μg), ofloxacin (5 μg), and tetracycline (30 μg). The results of antimicrobial susceptibility testing were interpreted following the guidelines of Clinical and Laboratory Standards Institute (Clinical and Laboratory Standards Institute [CLSI], 2018). The multiple antibiotic resistance (MAR) index of each strain was calculated by dividing the number of antibiotics to which a strains was resistant by the number of antibiotics that were tested (Krumperman, 1983). Here, the number of antibiotics to which an *E. coli* strain exhibited resistance was divided by 16 because susceptibility of each strain was tested for 16 antibiotics. The *E. coli* strains were tested for production of extended-spectrum β-lactamases (ESBLs) using a phenotypic confirmatory test recommended by the CLSI (Clinical and Laboratory Standards Institute [CLSI], 2018). Briefly, cefotaxime and ceftazidime disks (30 μg) alone and in combination with clavulanic acid (30/10 μg) were placed on the surface of bacterial lawn spread over Mueller–Hinton agar petri plates. Strains whose zone diameter in the presence of antibiotic–clavulanic acid combination was ≥5 mm were considered as ESBL producers. The strains were tested for phenotypic production of AmpC using AmpC E-test strips (bioMérieux Inc., MO, United States) following the manufacturer’s instructions. Strains that showed cefotetan/cefotetan + cloxacillin (CN/CNI) ratio of ≥8 were considered as AmpC producers (Bajaj et al., 2015).

### Detection and Analysis of Antimicrobial Resistance Genes

#### Detection of Genes Encoding β-Lactamases and Genetic Environment of *bla*_*CTX–M–*15_

Genes encoding β-lactamases and ESBLs, viz., *bla*_*TEM*_, *bla*_*SHV*_, and *bla*_*CTX–M*_, were detected by PCR amplification of the AMR genes using group-specific primers that amplified the internal coding regions of the genes (Dhanji et al., 2011; Bajaj et al., 2015). The presence of plasmid-encoded AmpC enzymes of the CMY types was determined by PCR amplification of the AMR gene using published primers (Pérez-Pérez and Hanson, 2002). The promoter region and genetic environment of *bla*_*CTX–M–*15_ were studied in *bla*_*CTX–M–*15_-positive *E. coli* strains by PCR amplification of the corresponding regions using the primers and methods described earlier (Saladin et al., 2002; Dhanji et al., 2011). The primers and the annealing temperatures for amplification of the *bla*_*TEM*_, *bla*_*SHV*_, and *bla*_*CTX–M*_ and genetic environment *of bla*_*CTX–M*_ are described in Table 1. The contents of the PCR mixture and methods for purification of the PCR amplicons and sequencing were the same as used for 16s rRNA gene sequencing. The nucleotide sequence homology was analyzed using the nucleotide BLAST (BLASTn) available at NCBI.

**TABLE 1:** Primers and PCR conditions for amplification of antimicrobial resistance genes, integrons and genetic environment of *bla*_*CTX–M*_.

Primers	Nucleotide sequence	Target genes	Amplicon size (bp)	Annealing temperature (°C)	References
TEM1-F TEM-1-R	5′-TCAACAGCGGTAAGATCCTTGA-3′ 5′-TGCAACTTTATCCGCCTCCA-3′	*bla*_*TEM*_	500	60	Bajaj et al., 2015
SHV-f SHV-r	5′-AAATGGATCTGGCCAGCG-3′ 5′-AGCAGCTGCCGTTGCGAA-3′	*bla*_*SHV*_	481	60	Bajaj et al., 2015
ISEcp1/U1 MA3	5′-AAAAATGATTGAAAGGTGGT-3′ 5′-ACYTTACTGGTRCTGCACAT-3′	IS*Ecp1, bla*_*CTX–M–*15_	900	48	Saladin et al., 2002
CTX-M ORF477	5′-CCGTTTCCGCTATTACAAAC-3′ 5′-CTGGGACCTACGTGCGCCCG-3′	*bla*_*CTX–M–*15_, *orf477*	1050	55	Dhanji et al., 2011
CMY-f CMY-r	5′-AACACACTGATTGCGTCTGAC-3′ 5′- CTGGGCCTCATCGTCAGTTA-3′	*bla*_*CMY*_	1,226	55	Pérez-Pérez and Hanson, 2002
RMTA-F RMTA-R	5′-CTAGCGTCCATCCTTTCCTC-3′ 5′-TTTGCTTCCATGCCCTTGCC-3′	*rmtA*	653	57	Yamane et al., 2007
RMTB-F RMTB-R	5′-GCTTTCTGCGGGCGATGTAA-3′ 5′-ATGCAATGCCGCGCTCGT AT-3′	*rmtB*	173	60	Yamane et al., 2007
RMTC-F RMTC-R	5′-CGAAGAAGTAACAGCCAAAG-3′ 5′-ATCCCAACATCTCTCCCACT-3′	*rmtC*	711	55	Yamane et al., 2007
ARMA-F ARMA-R	5′-ATTCTGCCTATCCTAATTGG-3′ 5′-ACC TATACTTTATCGTCGTC-3′	*armA*	315	46	Yamane et al., 2007
str-F str-R	5′-TATCTGCGATTGGACCCTCTG-3′ 5′-CATTGCTCATCATTTGATCGGCT-3′	*strA-strB*	538	62	Sunde and Norstrom, 2005
aacC2-F aacC2-R	5′-TAGAGGAGTATCGCGATGC-3′ 5′ATTATCATTGTCGACGGCCT-3′	*aacC2*	861	55	Ho et al., 2010
TetA-F TetA-R	5′-CAACAGACCCCTGATCGTAA-3′ 5′-AAAATTGCTTGCAGCGCC-3′	*tetA*	962	57	This study
TetM-F TetM-R	5′-ACAGAAAGCTTATTATATAAC-3′ 5′-TGGCGTGTCTATGATGTTCAC-3′	*tetM*	171	55	Aminov et al., 2002
TetW-F TetW-R	5′-GAGAGCCTGCTATATGCCAGC-3′ 5′-GGGCGTATCCACAATGTTAAC-3′	*tetW*	168	64	Aminov et al., 2002
QA-F QA-R	5′-TCGCCGCTGCCGCTTTTAT-3′ 5′-TTCGAGGTTGACCCGTCTG-3′	*qnrA*	517	60	Wang et al., 2009
QB-F QB-R	5′-AACCTGAAAGATGCCATT-3′ 5′-AAGGCCTTGTAAATCAAC-3′	*qnrB*	405	50	Wang et al., 2009
QC-F QC-R	5′-GGGTTGTACATTTATTGAATC-3′ 5′-TCCACTTTACGAGGTTCT-3′	*qnrC*	447	50	Wang et al., 2009
QD-F QD-R	5′-CGAGATCAATTTACGGGGAATA-3′ 5′-CGAGATCAATTTACGGGGAATA-3′	*qnrD*	582	57	Cavacoet al., 2009
QS-F QS-R	5′-GACGTGCTAACTTGCGTGAT-3′ 5′-GATCTAAACCGTCGAGTTCG-3′	*qnrS*	456	55	Bajaj et al., 2016
ACC-F ACC-R	5′TTGCGATGCTCTATGAGTGGCTA-3′ 5′-CTCGAATGCCTGGCGTGTTT-3′	*aac(6*’*)-Ib*	482	60	Chen et al., 2012
Int1-F Int1-R	5′-CCT CCC GCA CGA TGA TC-3′ 5′-TCC ACG CAT CGT CAG GC-3′	*intI1*	280	60	Kraft et al., 1986
hep58 hep59	5′-TCATGGCTTGTTATGACTGT-3′ 5′-GTAGGGCTTATTATGCACGC-3′	Variable region of class 1 integron	Variable	55	Whiteet al., 2000
qacE1-F sul1-R	5′-AAGTAATCGCAACATCCG-3′ 5′-GGGTTTCCGAGAAGGTGATTGC-3′	*qacEΔ1, sul1*	878	57	Bass et al., 1999; Nandi et al., 2004

#### PCR Amplification of Genes Encoding Plasmid-Mediated Quinolone Resistance, Aminoglycoside Resistance, and Tetracycline Resistance

The presence of genes encoding plasmid-mediated quinolone resistance (PMQR) was determined by PCR amplification of genes encoding for (i) proteins that protect DNA from quinolone binding (*qnr*A, *qnr*B, *qnr*C, and *qnr*D); (ii) *aac(6’)-Ib-cr* acetyltransferase (*aac*), which modifies fluoroquinolones like ciprofloxacin and enrofloxacin; and (iii) active efflux pump (*qep*A).

The presence of aminoglycoside resistance genes was determined by PCR amplification of the genes encoding for linked *str*A-*str*B genes and four types of plasmid-mediated 16S rRNA methylases—*arm*A, *rmt*A, *rmt*B, and *rmt*C— using published primers and annealing temperatures described in Table 1 (Sunde and Norstrom, 2005; Yamane et al., 2007).

The presence of tetracycline resistance genes was determined by PCR amplification of the tetracycline efflux gene *tet*A using self-designed primers and genes encoding ribosome protective proteins *tet*M and *tet*W using published primers and annealing temperatures (Aminov et al., 2002; Table 1). The contents of the PCR mixture and protocols for purification of the PCR amplicons, sequencing of PMQR, and aminoglycoside- and tetracycline-resistance genes and homology search were the same as used for the 16S rRNA genes.

### Detection and Analysis of Integrons and Gene Cassettes

The presence and distribution of integrase genes *intI1*, *intI2*, *intI3*, and integron class 1 gene cassette were determined by PCR amplification using published primers (Kraft et al., 1986; Goldstein et al., 2001; White et al., 2001). The variable regions (VRs) of class 1 integrons, which mainly contain an array of gene cassettes, are flanked at 3′ by a conserved segment containing qacEΔ1 and genes coding for quaternary ammonium and sulfonamide, respectively. VRs were investigated as previously reported in all isolates containing class 1 integrons (Guo et al., 2011). The PCR conditions for amplifying the VRs of integrons were the same as for amplifying the 16S rRNA gene except for the primers and annealing temperatures, which have been summarized in Table 1. The PCR amplicons were purified and sequenced as described for the 16S rRNA gene, and nucleotide sequence homology was analyzed using the nucleotide BLAST (BLASTn) available at NCBI.

### Accession Numbers

Gene sequencing revealed that the *bla*_*CTX–M–*15_ genes of the seven *bla*_*CTX–M–*15_-positive strains were identical to each other; hence, the partial coding sequence (CDS) of a representative strain (KP20) was submitted to GenBank (NCBI) with the accession number KF040057. The partial CDS of *bla*_*TEM–*1_ was also identical to each other; hence, the sequence of one representative strain (IP1N) was submitted to GenBank (NCBI) with the accession number KF055435. The partial CDS of *qnr*S was also identical; hence, the CDS of a representative strain (KP20) was submitted to NCBI GenBank under accession number KF055436. The partial CDS of *tet*A gene of all the three strains (IS47, WB3, and KKC) was submitted under the accession numbers KJ409940–KJ409942.

## Results and Discussion

### Molecular Identification and Phylogrouping Based on Triplex PCR

The results of 16S rRNA gene sequencing and homology search using BLAST confirmed that the strains presumptively identified using API 20E strips (bioMérieux, France) were *Escherichia coli*. The results of the triplex PCR and Clermont classification indicated that 50% (*n* = 20) of the strains belonged to phylogroup A [*chuA* (−), *yjaA* (−/+), and TSPE4.C2 (−)] while 50% (*n* = 20) to phylogroup B1 [*chuA* (−), *yjaA* (−), and TSPE4.C2 (+)]. Earlier studies have reported that *E. coli* strains of all phylogroups were present in river Yamuna (Bajaj et al., 2015; Kaushik et al., 2018). Phylogroups A and B1 of *E. coli* represent commensal strains, while phylogroups B2 and D represent pathogenic strains (Herzer et al., 1990; Bingen et al., 1998; Lecointre et al., 1998; Picard et al., 1999). Several studies have indicated that the prevalence of virulence genes in commensal strains of *E. coli* was lesser than in pathogenic strains (Johnson, 1991; Boyd and Hartl, 1998; Lecointre et al., 1998; Picard et al., 1999) but that the commensal strains can easily disseminate AMR determinants to pathogenic *E. coli* or other waterborne pathogens via mobile genetic elements. Thus, AMR determinants and integrons were investigated in these 40 commensal *E. coli* strains.

### Phenotypic Testing of Antimicrobial Susceptibilities and Extended-Spectrum β-Lactamase Production

Antibiotic susceptibility testing revealed that 95% (*n* = 38) of the commensal *E. coli* strains wereresistant to ampicillin, while 32% (*n* = 13) of the strains were resistant to piperacillin. Among the cephalosporins, 42.5% (*n* = 17) strains were resistant to cefazolin (first-generation cephalosporin), 17.5% (*n* = 7) to cefuroxime (second-generation cephalosporin), 22.5% (*n* = 9) to cefotaxime (third-generation cephalosporin), and 15% (*n* = 6) to cefepime (fourth-generation cephalosporin). The fact that commensal waterborne *E. coli* were less resistant to new-generation cephalosporins than ampicillin is normal because ampicillin was a widely prescribed broad-spectrum penicillin, and over time, bacteria might have developed resistance to this antibiotic. Earlier studies have also reported that ampicillin resistance was highly prevalent in commensal strains of *E. coli* isolated from India and from other parts of the globe like Vietnam, China, Sudan, and Thailand (Dyar et al., 2012; Abdelgader et al., 2018; Lugsomya et al., 2018; Singh A. K. et al., 2018; Purohit et al., 2019). With regard to ESBL production, 17.5% (*n* = 7) of the commensal *E. coli* strains tested positive, while none of the strain tested positive for AmpC production (Table 2). All the ESBL-producing strains were resistant to four or more β-lactam antibiotics, and 57% (*n* = 4) of the ESBL producers were resistant to ciprofloxacin. Since, penicillins and cephalosporins are the most frequently used antibiotics in India, it is normal that all the seven ESBL-producing *E. coli* strains were resistant to many antibiotics of these classes. The fact that some ESBL producers were also resistant to ciprofloxacin and streptomycin/kanamycin suggests that besides β-lactam antibiotics, resistance to other antibiotic classes also exhibited co-selection.

**TABLE 2:** Detailed information about the commensal strains of *E. coli* isolated from river Yamuna, antimicrobial resistance phenotypes, multiple antibiotic resistance index (MAR index) and antimicrobial resistance genes along with the genetic environment of *bla*_*CTX–M–*15_.

Strain designation (Phylogroup)	Antimicrobial resistance and ESBL^*a*^ phenotype	MAR^*b*^ index	Antimicrobial resistance genes	Genetic environment of *bla*_*CTX–M–*15_
KKC (A)	PIP, AMP, AMC, STM, TE	0.312	*bla*_*TEM–1*_, *tet*A, *str*A*-str*B*, qnrS1*	–
WB3 (A)	AMP,TE	0.125	*bla*_*TEM–1*_, *tet*A, *str*A*-str*B, *qnrS1*	–
NG23 (A)	AMP, NA	0.125	*bla*_*TEM–1*_, *arm*A	–
IS47 (A)	PIP, AMP, AMC, CTX, S, TE, ESBL	0.375	*bla*_*TEM–*1_, *bla*_*CTX–M–*15_, *tet*A, *str*A*-str*B, *qnr*S1	IS*Ecp1*, *orf477*
KP6 (A)	–	–	–	–
IPB (A)	AMP, NA	0.125	*bla*_*TEM–*1_	–
KK5 (A)	PIP, AMP, NA, CIP, OF, STM	0.375	*bla*_*TEM–*1_, *arm*A, *str*A*-str*B	-
KP20 (A)	PIP, AMP, AMC, CZ, CXM, CTX, CPM, STM, TE, ESBL	0.565	*bla*_*TEM–*1_, *bla*_*CTX–M–*15_, *arm*A, *str*A*-str*B, *qnrs*S1	IS*Ecp1*, *orf477*
ISF (A)	AMP, AMC, CZ, CXM, CTX, TE, TOB	0.437	*bla*_*TEM–1*_, *arm*A, *str*A*-str*B	–
NG35 (A)	AMP, NA, CIP, OF	0.25	*bla*_*TEM–1*_, *qnrS1*	–
WB2 (A)	PIP, AMP, AMC, CZ, STM	0.312	*bla*_*TEM–1*_, *arm*A, *qnr*S1	–
KK47 (A)	AMP	0.062	*bla*_*TEM–1*_	–
DND24 (A)	AMP, NA	0.125	*bla*_*TEM–1*_	–
IP1N (A)	PIP, AMP, AMC, CZ, CXM, CTX, CPM, NA, CIP, OF, TE, KAN, ESBL	0.75	*bla*_*TEM–*1_, *bla*_*CTX–M–*15_, *arm*A	IS*Ecp1*, *orf477*
KK1 (A)	AMP, CZ	0.125	*bla*_*TEM–1*_	–
NeG15 (A)	AMP, CZ	0.125	*bla*_*TEM–1*_	–
NG6 (A)	AMP, NA	0.125	*bla*_*TEM–1*_	–
ISJ (A)	AMP, CZ	0.125	*bla*_*TEM–1*_	–
KK26 (A)	AMP, CZ	0.125	*bla*_*TEM–1*_	–
PA18 (A)	AMP, CZ	0.125	*bla*_*TEM–1*_	–
NG41 (B1)	PIP, AMP, CZ, CXM, CTX, CPM, NA, CIP, ESBL	0.5	*bla*_*TEM–*1_, *bla*_*CTX–M–*15_	IS*Ecp1*, *orf477*
NG31(B1)	PIP, AMP, CZ, CXM, CTX, CPM, CIP, NA, ESBL	0.5	*bla*_*TEM–*1_, *bla*_*CTX–M–*15_	IS*Ecp1*, *orf477*
DND3(B1)	AMP, CZ, NA	0.187	*bla*_*TEM–1*_, *arm*A	–
KK15(B1)	AMP, CZ	0.125	*bla*_*TEM–1*_	–
PA1(B1)	AMP, CZ	0.125	*bla*_*TEM–1*_	–
KK21(B1)	AMP	0.062	*bla*_*TEM–1*_	–
PA3(B1)	PIP, AMP, TE	0.187	*bla*_*TEM–1*_	–
SVN(B1)	PIP, AMP, AMC, CZ, NA, CIP, OF, TE, TOB, KAN, NET, AK	0.75	*bla*_*TEM–1*_	–
MKNA(B1)	–	–	–	–
DND1(B1)	AMP, AMC	0.125	*bla*_*TEM–1*_	–
PA32(B1)	AMP	0.062	*bla*_*TEM–1*_	–
KK39(B1)	AMP, NA	0.125	*bla*_*TEM–1*_	–
IPK(B1)	AMP	0.062	*bla*_*TEM–1*_	–
WB20(B1)	AMP, CTX, TE	0.185	*bla*_*TEM–1*_	–
NG25(B1)	PIP, AMP, AMC, CZ, CXM, CPM, CTX, CIP, OF, NA, ESBL	0.625	*bla*_*TEM–*1_ *bla*_*CTX–M–*15_	IS*Ecp1*, *orf477*
DND11(B1)	AMP	0.062	*bla*_*TEM–1*_	–
IS68(B1)	AMP, TE	0.125	*bla*_*TEM–1*_	–
SP13N(B1)	PIP, AMP, AMC, CZ, CXM, CTX, CPM, ESBL	0.437	*bla*_*TEM–*1_, *bla*_*CTX–M–*15_, *qnr*S1	IS*Ecp1*, *orf477*
IS45(B1)	PIP, AMP, AMC, CIP, NA, OF, TE, TOB, KAN, NET, AK	0.685	*bla*_*TEM–1*_, *arm*A	–
WB9(B1)	AMP	0.062	*bla*_*TEM–1*_	–

*PIP, piperacillin; AMP, ampicillin; AMC, amoxicillin-clavulanic acid; CZ, cefazolin; CXM, cefuroxime; CTX, cefotaxime; CPM, cefepime; NA, nalidixic acid; CIP, ciprofloxacin; OF, ofloxacin; STM, streptomycin; TOB, tobramycin; KAN, kanamycin; NET, netilmycin; AK, amikacin; TE, tetracycline; ESBL^ a^: extended-spectrum β-lactamase; MAR^ b^ index: multiple antibiotic resistance index.*

With regard to quinolone resistance, 35% (*n* = 14) strains were resistant to older quinolones like nalidixic acid, while 20% (*n* = 8) and 15% (*n* = 6) strains were resistant to newer quinolones like ciprofloxacin and ofloxacin, respectively. In this regard, our results are similar to those of other studies that reported lower ciprofloxacin and ofloxacin resistance in waterborne *E. coli* isolated from other parts of the world (Odonkor and Addo, 2018). Aminoglycoside resistance was observably less prevalent with 12.5% (*n* = 5) strains resistant to older aminoglycosides like streptomycin and less than 7% strains resistant to new aminoglycosides like kanamycin, tobramycin, netilmicin, and amikacin. An earlier study also reported that a low level of aminoglycoside resistance was present in *E. coli* strains isolated from aquatic environments of Kuala Lumpur, Malaysia (Hara et al., 2018). With regard to tetracycline, 27.5% (*n* = 11) of the strains exhibited resistance. The frequent use/misuse of ampicillin, streptomycin, and tetracycline due to frequent prescription, availability, and affordability might be a probable reason for higher bacterial resistance to these antibiotics (Shakya et al., 2013). Additionally, 57.5% (*n* = 23) *E. coli* strains were resistant to at least two antibiotic classes, and 20% (*n* = 8) of the strains were multidrug resistant (MDR), i.e., resistant to three or more antibiotic classes. MDR *E. coli* were defined as bacteria resistant to antibiotics belonging to three or more antimicrobial classes (Magiorakos et al., 2012). An analysis of MAR index revealed that the MAR indexes of 13 MDR *E. coli* strains were quite high (>0.2). Tambekar et al. (2006) reported that bacteria isolated from environments where several antibiotics are used usually show MAR index >0.2. The high MAR index of the *E. coli* strains observed in this study is not surprising because river Yamuna is highly contaminated with effluents originating from hospital and municipal wastewaters; discharge from livestock, poultry, and agriculture production plants; etc.

### Antimicrobial Resistance Genes

#### β-Lactam Resistance and Extended-Spectrum β-Lactamase Encoding Genes

With regard to β-lactam resistance genes, *bla*_*TEM*–1_ was present in 95% of thestrains (*n* = 38) followed by *bla*_*CTX–M*–15_, which was present in 15% (*n* = 7) of the *E. coli* strains. The plasmid-encoded AmpC enzymes (CMY types) were not found in any strain. The presence of *bla*_*TEM–*1_ correlated well with ampicillin resistance in all the strains. Similarly, all the *bla*_*CTX–M–*15_-positive strains showed phenotypic production of ESBLs. Earlier studies had reported that *bla*_*TEM*–1_ was widely present in *E. coli* strains isolated from water bodies of India andother countries like Spain, Australia, France, China, and Poland (Lartigue et al., 2002; Tristram and Nichols, 2006; Garcia-Cobos et al., 2008; Ortega et al., 2012; Liu et al., 2014; Ojdana et al., 2014; Bajaj et al., 2015; Singh N. S. et al., 2018). CTX-M enzymes belong to the family of ESBLs and are the most widely disseminated ESBLs among Enterobacteriaceae all over the globe (Poirel et al., 2002). Among these, *bla*_*CTX–M–*15_ is the most widely globally disseminated CTX-M type, which was first reported from the Indian isolates in 2001 (Karim et al., 2001; Poirel et al., 2002). Later, several studies also reported the prevalence of *bla*_*CTX–M–*15_ in aquatic *E. coli* isolated from India (Bajaj et al., 2015; Singh N. S. et al., 2018; Kaushik et al., 2019). Previous studies have associated *bla*_*CTX–M–*15_ in aquatic *E. coli* with the pathogenic phylogroups B2 (especially those belonging to the genetic lineage ST131) and D (Nicolas-Chanoine et al., 2008; Coque et al., 2008). However, our study revealed that CTX-M-15 type ESBLs were present in the commensal phylogroups A and B1, also.

### Aminoglycoside Resistance Genes

The linked *str*A-*str*B genes are the most widely prevalent streptomycin resistance genes in *E. coli* worldwide and encode for phosphotransferases (Poirel et al., 2018). However, *str*A-*str*B genes were present in only 12% (*n* = 5) of the waterborne *E. coli*. Of these, four strains exhibited phenotypic resistance to streptomycin, while one strain (ISF), despite harboring *str*A-*str*B, was phenotypically susceptible for streptomycin. The 16S rRNA methylases methylate certain amino acid residues of the 16S RNA, resulting in resistance to amikacin, tobramycin, gentamicin, and netilmicin (Griffey et al., 1999). Of the four types of plasmid-mediated 16S rRNA methylase investigated, only *arm*A was found to be present in 17.5% (*n* = 7) of the strains. Earlier studies also reported that a low level of aminoglycoside resistance was present in *E. coli* strains isolated from aquatic environments in Kuala Lumpur, Malaysia (Hara et al., 2018). No correlation was observed in aminoglycoside resistance and presence of *arm*A, except in the *E. coli* strain IS45, which also exhibited phenotypic resistance to amikacin, tobramycin, kanamycin, and netilmicin.

### Plasmid-Mediated Quinolone Resistance and Tetracycline Resistance Genes

Of the several PMQR genes (*qnr*A, *qnr*B, *qnr*C, *qnr*D, *qnr*S, *qep*, and *aac*) tested, only *qnr*S was detected in the *E. coli* strains isolated from river Yamuna. The *qnr*S1 was detected as the predominant PMQR gene in about 17% of the aquatic strains (*n* = 7). Earlier studies have also reported that the *qnr*S type gene was the most frequently detected PMQR gene in *E. coli* isolated from environmental *E. coli* worldwide (Bonemann et al., 2006; Cattoir et al., 2008; Rodriguez-Mozaz et al., 2015; Varela et al., 2016; Hara et al., 2018). The presence of *qnr*S1 did not correlate with fluoroquinolone resistance, except in *E. coli* strain NG35. This suggests that the presence of the *qnr*S gene alone might not be a true indicator of fluoroquinolone resistance and the isolate despite that the presence of *qnr*S might exhibit phenotypic susceptibility for fluoroquinolones (Mahmud et al., 2020).

Of the three tetracycline resistance genes, *tet*M and *tet*W were absent and only *tet*A was present in 7.5% (*n* = 3) of the strains. Earlier studies have also indicated that tetracycline efflux-related genes like *tet*A, *tet*B, and *tet*C were more prevalent than ribosomal protection-related genes (like *tet*M and *tet*W) in waterborne *E. coli* (Zhang et al., 2015; Stange et al., 2016). The presence of *tet*A correlated well with phenotypic resistance because the three strains that harbored *tet*A also exhibited tetracycline resistance.

### Genetic Environment of *bla*_*CTX–M–*15_

Of the 40 *E. coli* strains investigated in this study, only seven strains (15%) harbored the *bla*_*CTX–M–*15_ gene. The upstream region of the *bla*_*CTX–M–*15_ was analyzed in these seven strains by PCR amplification and sequencing. Gene sequencing revealed that the IS*Ecp1* was present in the upstream region of *bla*_*CTX–M–*15_ and *orf477* was present in the downstream region of all the seven strains (Table 2). Several investigators have also reported the presence of *orf477* in the downstream region of *bla*_*CTX–M–*15_ (Eckert et al., 2006; Dhanji et al., 2011; Wang et al., 2014; Ben Said et al., 2016). IS*Ecp1* is the most common and widely reported IS element (Dhanji et al., 2011; Liu et al., 2014; Upadhyay et al., 2015; Ben Said et al., 2016; Singh N. S. et al., 2018). In the present study, IS sequence IS*Ecp1* was found to be present at 48-bp upstream region of *bla*_*CTX–M–*15_. The 42- to 266-bp upstream region has been reported as the preferred insertion site of IS*Ecp1* for different *bla*_*CTX–M*_ genes like CTX-M-1, CTX-M-2, and CTX-M-9. An analysis of the −35 and −10 promoter regions of the *bla*_*CTX–M–*15_ gene of the seven strains revealed that the −35 (TTGAAA) and −10 (TACAAT) regions were present within 3′ terminus end of IS*Ecp1* and 48 bp away from the *bla*_*CTX–M–*15_ start codon. The same organization was previously reported from *E. coli* strains isolated from different countries of the world (Saladin et al., 2002; Boyd et al., 2004; Canton and Coque, 2006; Lavollay et al., 2006; Dhanji et al., 2011; Liu et al., 2014). The presence of IS*Ecp1* along with *bla*_*CTX–M*_ has been reported from *E. coli* strains isolated from different parts of the world, indicating that IS*Ecp1* might be evolutionary associated with *bla*_*CTX–M*_ (Karim et al., 2001; Saladin et al., 2002; Dhanji et al., 2011; Liu et al., 2014; Wang et al., 2014). IS*Ecp1* can mobilize an adjacent gene as a part of transposition units of varying sizes (Zong et al., 2010). It has also been reported that IS*Ecp1* helps in improving the expression of *bla*_*CTX–M*_ in enteric bacteria (Poirel et al., 2003). This is a matter of great concern because the subsequent transfer of ESBL genes from these commensal *E. coli* to pathogenic *E. coli* or other bacteria in aquatic water bodies might pose a serious health challenge (Figueira et al., 2011).

### Detection and Analysis of Integrons

The class 1 integron gene *intI1* was detected in 75% (*n* = 30) of the isolates (Table 3). None of the strains harbored class 2 and 3 integrase genes *intI2* and *intI3*. Though some strains of *E. coli* reportedly harbored class 2 integrons, mostly class 1 integrons have been reported from *E. coli* isolated from India (Kaushik et al., 2018). Several studies have indicated that integrons of the class 3 were absent in *E. coli* isolated from water bodies across the globe (Laroche et al., 2009; Su et al., 2012; Pereira et al., 2013). Of the 30 *intI1* harboring strains, gene cassette arrays were detected in only 10% (*n* = 4) of the *intI1*-positive *E. coli* strains. Three different types of gene cassette arrays of class 1 were present in the downstream region of *intI1* whose size ranged from 1.7 to 2.8 kb (Table 2). Gene sequencing revealed that five gene cassettes of the dihydrofolate reductase (dfr) resistance gene family (*dhfr12* and *dfrA1*), aminoglycoside (*aad*) resistance gene family (*aadA1*, *aadA2*, and *aacA4*), and chloramphenicol (CHL) resistance gene family (*catB3*) were present (Table 3). The prevalence of class 1 integron *intI1* in Indian aquatic isolates was quite high (75%) and alarmingly more than that reported for *E. coli* isolated from global aquatic environments (Dolejska et al., 2009; Laroche et al., 2009; Pereira et al., 2013; Ghaderpour et al., 2015; Sidhu et al., 2017). *E. coli* isolates harboring class 1 integrons have been associated with a significantly higher probability for multidrug resistance than those devoid of class 1 integrons (Chen et al., 2011). Moreover, class 1 integron genes *intI1* are accompanied by resistance genes for disinfectants and heavy metals (Partridge et al., 2001) and can also easily horizontally transfer between strains originating from different sources (Nagachinta and Chen, 2008; Zhang et al., 2009). Thus, due to high prevalence of class 1 integron gene *intI1*, the commensal strains of *E. coli* can become vehicles for widespread dissemination of antibiotic resistance to pathogenic *E. coli* and other waterborne bacterial pathogens.

**TABLE 3:** Characteristics of integrons present in commensal *E. coli* strains isolated from river Yamuna, India.

*E. coli* strains	Class of integron gene	Size of variable gene cassettes (bp)	Gene cassette array
KKC	*intI1*	–	–
DND24	*intI1*	–	–
KP20	*intI1*	–	–
KK5	*intI1*	–	–
WB2	*intI1*	2,800	*aacA4, catB3, dfrA1*
KK26	*intI1*	1,700	*dfrA1, aadA1*
IPB	*intI1*	1,700	*dfrA1, aadA1*
KK1	*intI1*	–	–
PA18	*int1*	–	–
KP6	*intI1*	–	–
NG6	*intI1*	–	–
WB3	*intI1*	–	–
IP1N	*intI1*	–	–
NG35	*intI1*	–	–
NG23	*intI1*	–	–
PA3	*intI1*	–	–
NG41	*intI1*	–	–
NG31	*intI1*	–	–
MKNA	*intI1*	–	–
PA1	*intI1*	–	–
IS47	*intI1*	–	–
SVN	*intI1*	–	–
WB9	*intI1*	–	–
NG25	*intI1*	–	–
WB20	*int1*	1,900	*dhfr12, aadA2*
IS68	*intI1*	–	–
DND11	*intI1*	–	–
IS45	*intI1*	–	–
SP13N	*intI1*	–	–
KK39	*intI1*	–	–

## Conclusion

Our results indicated a high prevalence of drug resistance in *Escherichia coli* strains of river Yamuna. With regard to plasmid-mediated AMR genes, *bla*_*TEM–*1_ was present in 95% strains followed by q*nr*S1 and *arm*A (17% each), *bla*_*CTX–M–*15_ (15%), *str*A*-str*B (12%), and *tet*A (7%). Though most of the earlier studies have reported that *bla*_*CTX–M–*15_ in waterborne *E. coli* was mostly present in pathogenic phylogroup B2, our study revealed that CTX-M-15 type ESBLs were present in the commensal phylogroups A and B1, also. The genetic organization of *bla*_*CTX–M–*15_ was similar to that reported for *E. coli* globally, and IS*Ecp1* was present in the upstream region of *bla*_*CTX–M–*15_. Though integrons of classes 2 and 3 were absent, class 1 integron gene *intI* was detected in 75% of the isolates, which indicates its high prevalence in the *E. coli* isolates of the river Yamuna than that reported, globally. The presence of MDR phenotypes, plasmid-mediated AMR genes, and class 1 integron gene *intI1* in *E. coli* is a serious public health risk, because these commensal strains can become potent vehicles for widespread dissemination of AMR determinants to pathogenic *E. coli* and other waterborne pathogens. Thus, our study suggests an urgent need for regular surveillance and management of natural water bodies to curtail the spread of antibiotic resistance in microorganisms.

## Data Availability Statement

The datasets presented in this study can be found in online repositories. The names of the repository/repositories and accession number(s) can be found in the article/supplementary material.

## Author Contributions

NSS, NS, MK, and JV analyzed the data. NSS and NS wrote the manuscript. JV conceptualized and supervised. All authors contributed to the article and approved the submitted version.

## Conflict of Interest

The authors declare that the research was conducted in the absence of any commercial or financial relationships that could be construed as a potential conflict of interest.
